# Emergent Uterine Arterial Embolization Using N-Butyl Cyanoacrylate in Postpartum Hemorrhage with Disseminated Intravascular Coagulation

**DOI:** 10.1155/2017/1562432

**Published:** 2017-01-30

**Authors:** Soichiro Obata, Michi Kasai, Junko Kasai, Kazuo Seki, Zenjiro Sekikawa, Izumi Torimoto, Shigeo Takebayashi, Fumiki Hirahara, Shigeru Aoki

**Affiliations:** ^1^Perinatal Center for Maternity and Neonate, Yokohama City University Medical Center, Yokohama, Japan; ^2^Diagnostic Radiology, Yokohama City University Medical Center, Yokohama, Japan; ^3^Department of Obstetrics and Gynecology, Yokohama City University Hospital, Yokohama, Japan

## Abstract

Although it is widely accepted that uterine artery embolization (UAE) is an effective therapeutic strategy for postpartum hemorrhage (PPH), no consensus has been reached regarding the efficacy of UAE in patients with PPH with disseminated intravascular coagulation (DIC). This single-center retrospective cohort study included patients treated with UAE using NBCA for PPH between 2010 and 2015. The patients were divided into DIC and non-DIC groups, according to the obstetrical DIC score and the overt DIC diagnostic criteria issued by the International Society of Thrombosis and Haemostasis (ISTH), and their clinical outcomes were compared. There were 28 patients treated with UAE using NBCA. Complete hemostasis was achieved by UAE in 19 of 28 patients. In eight of nine patients with unsuccessful hemostasis, surgical hemostatic interventions were performed after UAE, and hemostasis was achieved in seven patients. UAE using NBCA showed no significant intergroup differences in complete hemostasis according to the presence or absence of DIC based on obstetrical DIC score (70% versus 62.5%, *P* = 1.000) or ISTH DIC score (54.5% versus 76.5%, *P* = 0.409). UAE using NBCA may be a useful first-choice treatment for PPH with DIC.

## 1. Introduction

Postpartum hemorrhage (PPH) remains one of the leading causes of maternal death [1], and it is widely accepted that uterine artery embolization (UAE) is an effective therapeutic strategy for PPH [2]. On the other hand, no consistent view has been established regarding the efficacy and safety of UAE in patients with PPH with unstable maternal hemodynamics or disseminated intravascular coagulation (DIC). In 2012, Lee et al. [3] reported that although embolization performed in 251 patients with PPH resulted in a clinical success rate of 86.5% (217/251 patients), the rate was lower in those with DIC. Similarly, Kim et al. [4] reported in 2013 that the clinical success rate was low in patients with DIC, 37.5% of whom developed complications.

In previous reports, an absorbable gelatin sponge is a commonly used embolic material [3–5], whereas few reports have described the use of N-butyl-2-cyanoacrylate (NBCA), which does not depend on maternal clotting factors [6, 7]. At our hospital, NBCA is actively used in cases of UAE performed for patients with PPH with clinical signs of DIC. In this study, cases of UAE performed with NBCA in patients with PPH with DIC were retrospectively analyzed to evaluate the safety and efficacy.

## 2. Materials and Methods

This single-center retrospective cohort study included patients both with DIC and without DIC treated with UAE using NBCA for PPH between 2010 and 2015. These cases of UAE performed with NBCA for PPH at our hospital were retrieved from our perinatal database. UAE using NBCA was performed in 28 patients. The obstetrical DIC score [8] (Table 1) was determined in each patient, and patients with a score ≥ 8 were diagnosed with DIC. The patients were also evaluated with the overt DIC diagnostic criteria issued by the International Society of Thrombosis and Haemostasis (ISTH) [9] (Table 1), and those with a score ≥ 5 were diagnosed with DIC (Table 1). According to each scoring system, the patients were divided into DIC and non-DIC groups, and their baseline characteristics and clinical outcomes were compared. Primary PPH was defined as PPH occurring within 24 hours after delivery, while secondary PPH was defined as PPH occurring at or beyond 24 hours after delivery. Initially, the patients of PPH were managed by administration of uterine contraction agent, such as oxytocin or methylergometrine, and surgical intervention, if necessary, such as repairing genital tract lacerations, uterine compression suture, hysterectomy, or manual extraction placenta. UAE was performed after consultations with interventional radiologists when the obstetricians determined that hemostasis was difficult to achieve by obstetric management. UAE was performed even for patients with hemodynamic instability as well as those with hemodynamic stability.

UAE was performed by two board-certified radiologists with 18 and 8 years of embolization experience. A 5-F shepherd hook catheter (Catex-CX, Gadelius Medical Co., Tokyo, Japan) or a 4-F cobra-head catheter (Terumo Co., Tokyo, Japan) was used. For the selective catheterization of the uterine artery, a 2.1-F microcatheter (Sniper 2, Terumo Co.) or 2.0-F high flow microcatheter (Bobsled, Clinical Supply Co., Yokohama, Japan) was passed through the parent catheter with a 0.016-inch microguidewire (Double Angle-Radifocus, Terumo Co.). NBCA (Histoacryl, B. Braun Melsungen, Germany) mixed with iodized oil (Lipiodol, Guerbet, Tokyo, Japan) at a ratio of 1 : 4 (20%) was usually used. Following a flush of 20% glucose, a 2.5 mL syringe containing 2.5 mL of 20% glue was connected to the microcatheter for the single-column injection technique. In the embolization of the internal iliac main trunk, the glue was infused through a 2.4-F coaxial catheter, of which the tip was placed in the anterior trunk and gradually pulled back in the main trunk with continuous infusion. The patients were admitted to the intensive care unit after the UAE and treated with antibiotics for ≥3 days. The regimens of antibiotics were determined by the attending obstetrician based on each patient's condition.

The institutional perinatal database was used for a retrospective review of the included women's medical charts. All patients were divided into the DIC cohorts and the non-DIC cohorts according to each obstetrical DIC scoring system and the ISTH DIC scoring system (Table 1). Patients' backgrounds were compared between the DIC cohorts and the non-DIC cohorts each classified by the scoring system according to the following variables: the mean values of age, gestational age, time to UAE, procedure time of UAE, the volume of hemorrhage, the volume of each transfused fresh frozen plasma (FFP), and red blood cell (RBC). We also compared the incidences of patients with primipara, those with primary postpartum hemorrhage, those who underwent cesarean delivery, maternal transport in postpartum period, extravasation, and those with massive hemorrhage which was defined as a blood loss ≥ 2 L. The volume of blood loss was defined as the total volume of blood loss during a period from the delivery to the end of UAE. The effect of UAE was evaluated with regard to both complete hemostasis and efficacy of hemostasis. Complete hemostasis was defined as hemostasis achieved by UAE alone and requiring no additional hemostatic interventions. The efficacy of hemostasis was determined according to the number of cases of complete hemostasis and cases in which patients showed a decrease in DIC scores and underwent surgical intervention resulting in successful hemostasis. The time to UAE was defined as the time from a point when attending obstetricians recognized the need of UAE to the start of UAE in patients delivering at our hospital; in women transferred from other hospitals during the puerperal period, it was defined as the time from when obstetricians attending the delivery determined the transfer of their patients to our hospital for PPH control to the start of UAE. Furthermore, we also compared the frequencies of those with sepsis after UAE between the two patients groups. Sepsis was diagnosed in patients with positive blood cultures for samples obtained when a fever with a body temperature of ≥38°C developed after UAE.

This study was approved by the ethics committee of the Yokohama City University Medical Center (D1602001). We confirmed that the patients or the legal representatives of the patients in this study were given a comprehensive written statement of information about the clinical study, including information on UAE with NBCA-Lipiodol mixture, and their consent was documented in the clinical records.

IBM SPSS 23 statistical software (SPSS, Inc., Chicago, IL, USA) was used for the statistical analyses. The Mann–Whitney *U* test was used to analyze continuous variables, and Fisher's exact test was used to analyze intergroup differences in categorical data. The level of statistical significance was set at *P* < 0.05.

## 3. Results

**Figure 1:**
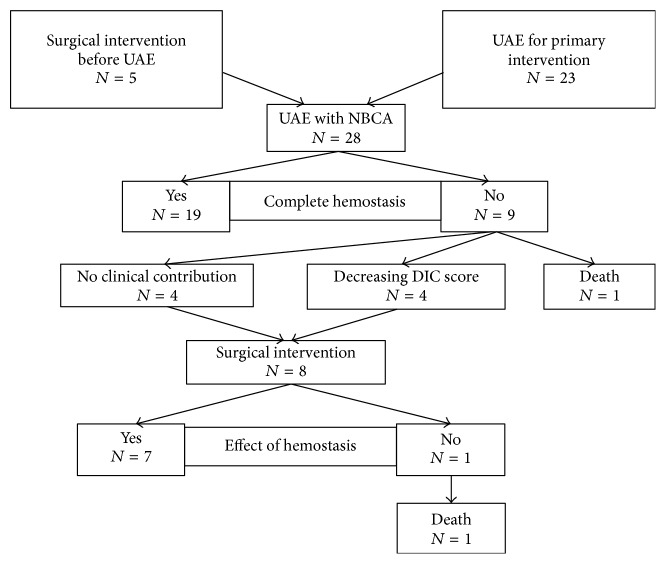


A flow chart of this study is shown in Figure 1. There were 28 patients treated with UAE using NBCA. The patients are summarized in Table 2 in terms of surgical intervention before UAE, embolized vessels, and presence or absence of DIC and extravasation. Of these patients, a surgical hemostatic intervention was first attempted in five patients, abdominal total hysterectomy (ATH) was attempted in three cases, and uterine compression suture and manual extraction of placenta were each attempted in one case, while UAE was first attempted in 23 patients. 18 patients in whom hypotension or tachycardia showed no response to the fluid resuscitation were transported directly to the angiographic suite in the obstetric emergency department. An indwelling intra-aortic balloon occlusion catheter was required in two of the 18 patients to maintain the blood pressure. In the remaining 10 patients, four patients were evaluated by contrasted enhanced computed tomography, and 3 patients showed extravasation and one patient showed pseudoaneurysm in uterus. Remaining 6 patients were not evaluated by computed tomography to avoid contrast material induced renal failure in the patients who had to be given contrast material on the UAE.

Complete hemostasis was achieved by UAE in 19 of 28 patients. In one of 9 patients with unsuccessful hemostasis, ATH was performed before UAE, and hemorrhage was difficult to control despite UAE, resulting in death. In the remaining 8 patients, surgical hemostatic interventions were performed after UAE. In four of these 8 patients, the obstetric DIC scores were decreased by UAE, whereas UAE made no clinical difference in the other four patients. The surgical hemostatic interventions performed included ATH in four patients, dilatation and curettage for a retained placenta in one patient, and laparotomy or transvaginal hemostatic intervention in three patients. Although one patient undergoing ATH died, hemostasis was achieved in the remaining seven patients who survived.

The results of comparison between the DIC and non-DIC groups according to the obstetrical DIC score are shown in Table 3. Twenty patients (71.4%) scored ≥8 points and were diagnosed with DIC. Although the gestational age was higher in the non-DIC group, no differences were observed in other variables, namely, maternal age, primipara rate, cesarean delivery rate, proportion of patients with primary PPH, proportion of patients transferred from other hospitals, time to UAE, procedure time of UAE, and presence or absence of extravasation. Complete hemostasis was achieved by UAE alone in 14/20 patients (70%) in the DIC group and 5/8 patients (62.5%) in the non-DIC group, although the difference between the groups was not significant (*P* = 1.000). The efficacy of hemostasis was confirmed in 17/20 (85%) and 6/8 (75%) patients, respectively, which also did not differ (*P* = 0.606). In maternal mortality, which occurred in 2/20 patients (10%) in the DIC group and 0/8 patients (0%) in the non-DIC group, no statistically significant difference was observed (*P* = 1.000). There were no significant differences in blood loss volume, blood transfusion volume, or maternal sepsis incidence.

**Figure 2:**
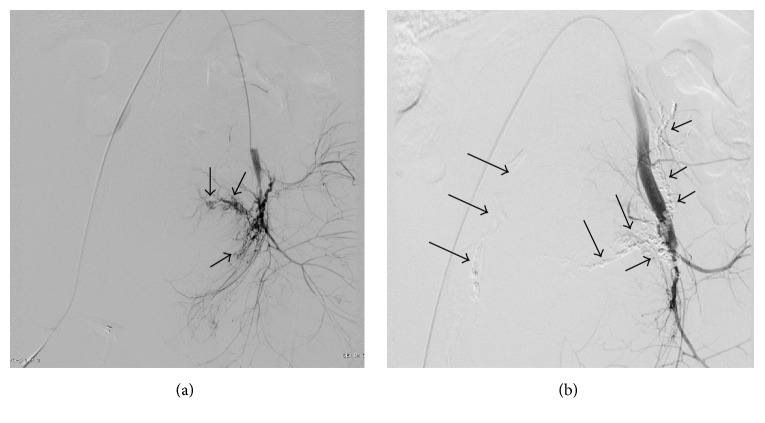
A 34-year-old patient with postpartum hemorrhage due to incomplete uterine rupture who had overt disseminated intravascular coagulation according to both the obstetrical DIC and the ISTH DIC scoring systems. (a) Left internal iliac artery angiography showing dilated intrauterine arteries and extravasation (arrows). (b) Left internal iliac angiography after embolization using a 20% N-butyl-2-cyanoacrylate-Lipiodol mixture shows glue cast (arrows) and complete obstruction of the uterine artery. Right uterine artery was also embolized by using N-butyl-2-cyanoacrylate-Lipiodol mixture.

The results of the comparison of the DIC and non-DIC groups according to ISTH DIC score are shown in Table 4. Eleven patients (39.3%) scored ≥5 points and were diagnosed with DIC. Regarding maternal characteristics, no differences were observed in maternal age, gestational age, primipara rate, cesarean delivery rate, proportion of patients with primary PPH, proportion of patients transferred from other hospitals, time to UAE, or presence or absence of extravasation. A case of overt DIC in which complete hemostasis was achieved with UAE using NBCA is shown in Figures 2(a) and 2(b). Complete hemostasis was achieved with UAE alone in 6/11 patients (54.5%) in the DIC group and in 13/17 patients (76.5%) in the non-DIC group, although the difference between the groups was not significant (*P* = 0.409). The efficacy of hemostasis was confirmed in 8/11 (72.7%) and 15/17 (88.2%) patients, respectively, the difference between which was not significant (*P* = 0.353). In maternal mortality, which occurred in 2/11 patients (18.2%) in the DIC group and 0/17 patients (0%) in the non-DIC group, no statistically significant difference was observed (*P* = 0.146). Although the doses of fresh frozen plasma and red blood cells were higher in the DIC group (*P* < 0.001 and *P* = 0.002, resp.), no differences were observed in blood loss volume or maternal sepsis incidence.

There were no cases of complication related to UAE such as uterine necrosis, distal embolization, and hematoma at punctual site.

## 4. Discussion

UAE using NBCA performed for PPH achieved complete hemostasis in 19/28 patients (67.9%) and exhibited hemostasis efficacy in 23/28 patients (82.1%). This procedure demonstrated therapeutic efficacy. Moreover, its efficacy as indicated by complete hemostasis and efficacy of hemostasis did not differ between groups according to obstetrical DIC score and ISTH DIC score. The survival rate was 93% (26/28 patients). In the two cases of maternal death, both patients had developed an amniotic fluid embolism before transfer to our hospital, and irreversible DIC was observed on their arrival. One patient who presented with near-cardiopulmonary arrest on her arrival underwent UAE before surgical intervention, while another patient underwent ATH before UAE. However, hemostasis was not achieved in either case, and both patients died.

UAE using NBCA showed no significant intergroup differences in complete hemostasis or efficacy according to the presence or absence of DIC based on obstetrical DIC score or ISTH DIC score. This procedure was effective in patients with PPH with DIC. PPH often rapidly leads to DIC. Thus, not only the commonly used ISTH DIC score but also the obstetrical DIC score, which rates severity according to clinical symptoms, is used for treatment decisions. In this study, all patients diagnosed with DIC according to ISTH DIC score were also diagnosed with DIC according to obstetrical DIC score, and no intergroup differences in therapeutic efficacy were observed according to either DIC score. In general, if coagulopathy is present, it should be corrected before the procedure. In particular, elective interventional procedures are considered relatively contraindicated under coagulopathy. In fact, two series demonstrated that DIC was a risk factor for failure of embolization to control hemorrhage [3, 10]. Lee et al. [3] reported that the success rates of interventional radiology were 86.4% (217/251 patients) for PPH and 62.5% (20/32 patients) for PPH complicated by overt DIC, while Cheong et al. [10] reported that the success rates were 88.0% (103/117 patients) and 75.8% (25/33 patients), respectively. They reported that hemostasis rates were lower in patients with DIC. However, in these previous reports, they prefer to choose absorbable gelatin sponge as embolization material, while we prefer to use NBCA, which is immediate and permanent embolic agent and its embolic effect can be achieved independent of patients' clotting function. The hemostasis rates in our study are comparable with those in these reports. However, in our study, UAE was the first choice even for patients with a hemodynamically and hemostatically unstable condition, while UAE was used for patients with a more severe condition than those described in their reports. Although this might have been responsible for the lower overall response rates as indicated by complete hemostasis achieved in 19/28 patients (67.9%) and efficacy of hemostasis observed in 23/28 patients (82.1%) in our study, our study did not show any differences in the hemostasis rate regardless of the presence or absence of DIC. Thus, our findings suggest that UAE with NBCA is highly effective for hemostasis in patients with PPH accompanied by DIC and might be a first-choice therapeutic strategy, especially for patients requesting fertility preservation.

This study has several limitations. First, because it included many patients who delivered babies at other hospitals, different protocols were used to treat PPH and the clinical courses prior to arrival at our hospital varied substantially among the patients. Moreover, even in patients whose treatment of PPH started at our hospital, decisions on which procedure, surgical interventions, or UAE was prioritized were left to each obstetrician's discretion, and the obstetricians did not follow the same protocol. Finally, the sample size of 28 was small.

UAE using NBCA has its disadvantages including higher costs compared to UAE using conventional embolic materials and the need for operators to accurately determine the occlusion site [11]. Moreover, although it has been reported that pregnancy after UAE using NBCA follows a normal course and results in a normal delivery [7], the impact of the procedure on a subsequent pregnancy is still unknown. However, the response rate achieved by UAE with NBCA for PPH with coagulopathy was comparable with the corresponding rate for PPH without coagulopathy.

## 5. Conclusion

Emergency UAE using NBCA may be a useful first-choice treatment for PPH with DIC.

## Figures and Tables

**Table 1:** Obstetrical DIC and ISTH DIC scores.

	Obstetrical DIC score	ISTH DIC score
Clinical background (abruptio placentae, amniotic fluid embolism, postpartum hemorrhage with DIC, or eclampsia)	1–5	—
Clinical signs (acute renal failure, acute respiratory failure, severe organ damage, bleeding tendency, or shock state)	0–34	—
Laboratory data	0–7	0–8

Diagnosis of DIC	≥8	≥5

DIC, disseminated intravascular coagulation; ISTH, International Society of Thrombosis and Haemostasis.

**Table 2:** The condition of DIC, surgical intervention before UAE, and findings of UAE in the individual cases.

Case	ISTH DIC	Obstetrical DIC	Surgical intervention before UAE	Extravasation	Embolized vessels
1	−	−	−	−	Uterine arteries, bilateral
2	−	−	Uterine compression suture	−	Uterine arteries, bilateral
3	−	+	−	−	Uterine arteries, bilateral
4	−	+	−	−	Uterine arteries, bilateral
5	−	+	Hysterectomy	+	Uterine arteries, bilateral
6	+	+	−	+	Uterine arteries, bilateral
7	−	+	−	−	Uterine arteries, left
8	−	−	−	−	Uterine arteries, bilateral
9	+	+	Hysterectomy	+	Internal iliac arteries, bilateral
10	+	+	−	+	Uterine arteries, bilateral
11	−	+	−	−	Uterine arteries, bilateral
12	+	+	−	−	Internal iliac arteries, bilateral
13	−	+	−	+	Uterine arteries, bilateral
14	+	+	−	−	Uterine arteries, bilateral
15	+	+	Manual extraction of placenta	−	Uterine arteries, bilateral
16	−	−	−	−	Uterine arteries, bilateral
17	+	+	−	+	Uterine arteries, bilateral
18	−	+	−	−	Uterine arteries, left
19	+	+	−	+	Uterine arteries, bilateral
20	−	+	−	+	Uterine arteries, bilateral
21	−	−	−	+	Uterine artery and internal pudendal artery, right
22	−	−	−	+	Internal pudendal artery, right
23	−	−	−	+	Internal pudendal artery, right
24	−	−	−	+	Uterine arteries, bilateral
25	+	+	−	+	Internal iliac arteries, bilateral
26	+	+	−	+	Uterine arteries, bilateral
27	−	+	Hysterectomy	+	Uterine arteries, bilateral
28	+	+	−	+	Gastroepiploic artery and cervicovaginal artery, right

**Table 3:** Comparison of DIC cohorts and non-DIC cohorts classified by obstetric DIC score.

	DIC (*N* = 20)	Non-DIC (*N* = 8)	*P* value
Age	34 (24–42)	31 (28–36)	0.11
Primipara	12 (70.6)	5 (62.5)	1
Gestational age (wks)	39.1 (34.9–41.1)	40.6 (37.1–42.0)	0.049^*∗*^
Cesarean delivery	7 (35.0)	1 (12.5)	0.371
Primary PPH	18 (90)	7 (87.5)	1
Maternal transport in postpartum period	16 (80.0)	6 (75.0)	1
Time to UAE (min)	112.5 (55–283)	105 (70–330)	0.784
Procedure time of UAE (min)	76 (46–130)	60 (32–118)	0.099
Hemorrhage (mL)	4500 (700–10578)	3300 (2000–6000)	0.381
Massive hemorrhage * *(≥2000 mL)	17 (85.0)	8 (100)	0.536
Extravasation	12 (60)	4 (50)	0.691
Complete hemostasis	14 (70.0)	5 (62.5)	1
Efficacy of hemostasis	17 (85.0)	6 (75.0)	0.606
Mortality	2 (10.0)	0 (0.0)	1
Transfusion of FFP (mL)	1800 (720–12240)	1080 (720–3120)	0.123
Transfusion of RBC (mL)	2160 (720–15840)	1320 (480–3600)	0.07
Sepsis	1 (5.0)	0 (0.0)	1

^*∗*^Significant difference.

DIC, disseminated intravascular coagulation; PPH, postpartum hemorrhage; UAE, uterine arterial embolization; FFP, fresh frozen plasma; RBC, red blood cells.

**Table 4:** Comparison of DIC cohorts and non-DIC cohorts classified by ISTH DIC score.

	DIC (*N* = 11)	Non-DIC (*N* = 17)	*P* value
Age	34 (29–42)	32 (24–40)	0.053
Primipara	6 (54.5)	11 (64.7)	0.701
Gestational age (wks)	38.4 (34.9–41.1)	40.0 (36.1–42.0)	0.208
Cesarean section	4 (36.4)	4 (23.5)	0.671
Primary PPH	11 (100.0)	14 (82.4)	0.258
Maternal transport in postpartum period	10 (90.9)	12 (70.6)	0.355
Time to UAE (min)	122 (65–283)	103 (55–330)	0.853
Procedure time of UAE (min)	53 (32–84)	77 (45–130)	0.017^*∗*^
Hemorrhage (mL)	4500 (1740–8000)	3550 (700–10578)	0.853
Massive hemorrhage (≥2000 mL)	10 (90.9)	15 (88.2)	1
Extravasation	8 (72.7)	8 (47.1)	0.253
Complete hemostasis	6 (54.5)	13 (76.5)	0.409
Efficacy of hemostasis	8 (72.7)	15 (88.2)	0.353
Mortality	2 (18.2)	0 (0.0)	0.146
Transfusion of FFP (mL)	4080 (1440–12240)	960 (720–3840)	<0.001^*∗*^
Transfusion of RBC (mL)	2880 (1200–15840)	1440 (480–3840)	0.002^*∗*^
Sepsis	1 (9.1)	0 (0.0)	0.393

^*∗*^Significant difference.

DIC, disseminated intravascular coagulation; PPH, postpartum hemorrhage; UAE, uterine arterial embolization; FFP, fresh frozen plasma; RBC, red blood cells.
